# Mastermind-Like 1 Is Ubiquitinated: Functional Consequences for Notch Signaling

**DOI:** 10.1371/journal.pone.0134013

**Published:** 2015-07-30

**Authors:** Mozhgan Farshbaf, Mikael J. Lindberg, Anh Truong, Zachery Bevens, Elaina Chambers, Angeliki Pournara, Annika E. Wallberg, J. Brandon White

**Affiliations:** 1 From the Department of Biological Sciences, San José State University, San José, California, United States of America; 2 From the Institute of Environmental Medicine, Karolinska Institutet, Stockholm, Sweden; San Diego State University, UNITED STATES

## Abstract

Early studies demonstrated the involvement of ubiquitination of the Notch intracellular domain for rapid turnover of the transcriptional complex at Notch target genes. It was shown that this ubiquitination was promoted by the co-activator Mastermind like 1 (MAML1). MAML1 also contains numerous lysine residues that may also be ubiquitinated and necessary for protein regulation. In this study, we show that over-expressed MAML1 is ubiquitinated and identify eight conserved lysine residues which are required for ubiquitination. We also show that p300 stimulates ubiquitination and that Notch inhibits ubiquitination. Furthermore, we show that a mutant MAML1 that has decreased ubiquitination shows increased output from a HES1 reporter gene assay. Therefore, we speculate that ubiquitination of MAML1 might be a mechanism to maintain low levels of the protein until needed for transcriptional activation. In summary, this study identifies that MAML1 is ubiquitinated in the absence of Notch signaling to maintain low levels of MAML1 in the cell. Our data supports the notion that a precise and tight regulation of the Notch pathway is required for this signaling pathway.

## Introduction

**Fig 1:**
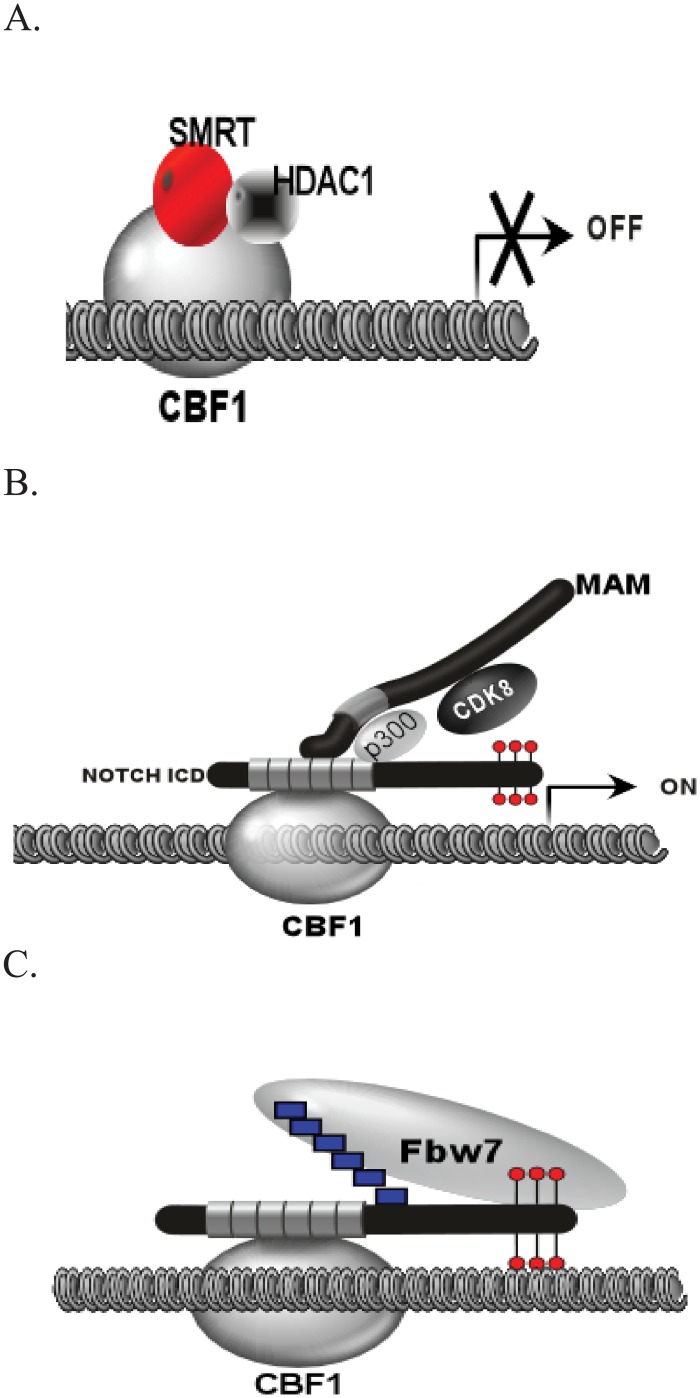
Overview of Notch Signaling in the Nucleus. (A) In the absence of the NICD, Notch target genes remain in a repressed state through interaction of CBF1 with corepressor complexes (SMRT and HDAC1). (B) Release of the NICD from the cell membrane results in nuclear translocation and recruitment of MAML1, p300, and CDK8. CDK8 phosphorylates the NICD in the PEST domain as indicated by the lollipop structures. (C) Phosphorylation is thought to recruit the ubiquitin ligase Fbw7 to poly-ubiquitinate (boxes) the NICD, thereby signaling for degradation and shut off of target gene activation.

The Notch signaling pathway is an evolutionarily conserved system found in all metazoans and it plays an important role in developmental and disease processes by influencing proliferation, differentiation, self-renewal and apoptosis [1, 2]. In mammals, there are four Notch receptors. The Notch receptor is a protease-activated transcription factor located on the cell membrane. Interaction with a ligand results in cleavage releasing the intracellular domain (NICD). The NICD enters the nucleus and forms a complex with CSL (CBF1, Suppressor of Hairless, and Lag-1) enhancer-binding proteins and MAML1 to activate target genes. The ternary complex (CSL, NICD, and MAML1) is believed essential for activation of target genes (Fig 1). MAML1 was initially identified as a protein involved in the Notch pathway in *D*. *Melanogaster* [3, 4]. In mammals, there are three family members (MAML1-3) [5]. The most well characterized interactions have been shown between Notch 1 (N1ICD) and MAML1.

MAML1 has been shown to play a dual role in Notch-dependent gene regulation in the ternary complex. First, MAML1 helps recruit p300 to target genes to acetylate histones [6, 7]. Second, MAML1 has been shown to interact with and recruit CDK8 to chromatin [8]. CDK8 has been shown to phosphorylate the PEST domain of NICD. This in turn is believed to recruit FBW7 to ubiquitin the N1ICD causing degradation of the N1ICD and shutoff of the target gene. FBW7 has previously been shown to interact and ubiquitinate the N1ICD [9, 10]. In addition, CDK8 has been shown to inhibit Notch acetylation and Notch transcription enhanced by p300 [11]. This shut off of signaling is believed to be important to reset target genes for further rounds of activation and help establish a gradient of notch signaling as has been previously shown during somitogenesis [12].

The rapid timing of Notch signaling requires strict control on the levels of free N1ICD in the nucleus to prevent aberrant transcriptional activation. Mutations in the PEST domain of the N1ICD have been shown to be important in stabilizing the N1ICD in various cancers. As a result, constitutive cMYC expression has been shown to drive cell division in these cancers [13, 14]. Further, proteolysis of N1ICD may correlate with attenuation of Notch activation of target genes thereby tuning the levels of N1ICD present and subsequent levels of gene expression [15, 16].

More recently, MAML1 has been shown to be a more general coactivator of transcription [17]. MAML1 has been shown to play important coactivator roles for p53, MEF2C, and EGR1 [18–20]. However, MAML1’s interaction with N1ICD may be the primary target as MAML1 has been shown to prefer interaction with N1ICD over MEF2C [19]. Therefore, understanding how MAML1 coordinates transcription between different transcriptional pathways is of extreme importance.

Modification of transcriptional activators has been shown to be important in fine-tuning the transcriptional output of the target genes. In addition to N1ICD phosphorylation and ubiquitination, numerous transcription factors have also been shown to be modified including cMYC and p53 (reviewed in [21]). The modifications have been shown to be important in regulating the transcriptional activity of these various factors. We have shown that MAML1 can be post-translationally modified [22, 23] and therefore we looked to see if MAML1 could be ubiquitinated. Using biochemical approaches, we show here that MAML1 is ubiquitinated and go on further to characterize the ubiquitination and its importance in activation of Notch target genes. We find that interaction with the N1ICD prevents ubiquitination. We postulate that MAML1 levels must remain low in the nucleus to prevent spurious activation of genes.

## Materials and Methods

### Plasmids

Human–MAML1, MAML2, MAML3, and CBF1 were cloned into pCS2 containing an N-terminal 6X-MycTag using Gateway technology and standard PCR. Deletion mutants of MAML1 (1–939, 1–710, 1–579, 1–478 and 1–301) were prepared using standard PCR and cloned into pCS2. MAML1 K/R (K112, 178, 188, 189, 405, 407, 639, and 822) was generated by site-directed mutagenesis using QuickChangeMulti (Agilent Genomics, Santa Clara, CA, USA). MAML1 and MAML1 K/R were also sub-cloned into p3X-FLAG vector from SIGMA-ALDRICH (St. Louis, MO, USA) for immunocytochemistry. The heme-agglutinin tagged ubiquitin (HA-Ub) expression plasmid was a kind gift of Dr. Dirk Bohmann (University of Rochester Medical Center). pCI-FLAG-p300 and pCI-FLAG-p300ΔHAT were kind gifts from Dr. J. Boyes (University of Leeds, UK).

### Antibodies

Anti-Myc (9E10), Anti- Glyceraldehyde 3-phosphate dehydrogenase (GAPDH) (FL-335), and Goat Anti-Mouse FITC conjugated antibodies were purchased from Santa Cruz Biotechnology (Santa Cruz, CA, USA). Antibody to FLAG tag was purchased from Pierce (Thermo Fisher Scientific, Rockford, IL, USA).

### Reporter Gene Assays

HeLa cells were transiently transfected with 100 ng HES1-Luc reporter, 10 ng pCS2-N1ICD, 100 ng pCS2-MAML1, and 20ng pRL-TK in a 96-well plate. The HES1-Luciferase reporter has been described previously [24]. Cells were harvested after 40–48 h and the levels of luciferase were measured with the Dual Luciferase Assay System from Promega on a GloMax-96 or GloMax-Multi+ Luminometer from Promega (Madison, WI, USA). The error bars on graphs represent standard deviation of three independent experiments.

HeLa cells were transiently transfected with 100 ng HES1-Luc reporter, 10 ng pCS2-Notch1 ICD, 100ng FLAG-MAML1 or FLAG-MAML1K/R and 20 ng pRL-TK in a 96-well plate. Cells were harvested after 40–48 h and the levels of luciferase were measured with the Dual Luciferase Assay System from Promega on a GloMax-96 or GloMax-Multi+ Luminometer from Promega (Madison, WI, USA). The error bars on graphs represent standard deviation of three independent experiments.

HeLa cells were cotransfected with 100 ng pG5-luc reporter and 40 ng GAL4-N1 ICD, 100 ng p300-HA, 100 ng CDK8-FLAG and 150 ng MAML1 plasmids using TransIT-LT1 transfection reagent (Mirus, Madison, WI, USA). After 48 h, the cells were harvested and luciferase activity was measured using LucySoft3 (Anthos Labtec, Salzburg, Austria). The bars represent standard deviations of three replicate samples.

### Immunfluorescent staining

25,000 HeLa cells were seeded into Lab Tek II 8 well chamber slide system. Twenty-four hours later, cells were transfected with 1.2 ug MAM1 WT or MAM1 K/R expression plasmid in Opti-MEM using Lipofectamine 2000 following recommended protocol. Twenty-four hours later, the media was replaced with DMEM cell culture media containing either 10 μM lactacystin or DMSO for an additional 24 hours. Cells were fixed and permeabilized using 4% Formaldehyde in PBS and 0.1% Triton X-100. Nonspecific binding sites were blocked with 5% nonimmune goat serum (GS) in PBS for 1 hour. Anti-FLAG epitope tag antibody was diluted 1:200 in PBS + 5% GS and applied to the cells for two hours at room temperature, washed extensively in PBS + 0.1% Triton X-100, and then incubated for 1 hour with secondary goat anti-mouse FITC conjugated secondary antibody. Cells were extensively washed with PBS + 0.1% Triton X-100 and then 2 mg/mL of Invitrogen’s Hoechst 33258 staining solution was applied 30 minutes, washed extensively in PBS and cells mounted with Vectashield mounting media (Vector Laboratories, Burlingame, CA). Cellular imaging was carried out using the Leica DMI 4000B florescent microscope with Leica Application Suite version 4.1.0. Excitation filter BP 360/40 and BP 480/40 was used to visualize Hoechst and FITC stains respectively.

### Chromatin assembly and in vitro transcription assay

The plasmid containing 12 binding sites for CSL was assembled into chromatin by using purified recombinant Drosophila Acf-1, ISWI, and NAP1 proteins as described previously[25]. For the transcription reactions, 70 ng of the chromatin-assembled template was preincubated with 10 ng N1ICD, 50 ng CSL, 100 ng MAML1, 100 ng p300, 100 ng CDK8 and 3 μM acetyl-CoA as indicated. Approximately 50 μg HeLa nuclear extract was then added per reaction and transcription was initiated by addition of 0.4 mM NTPs. For primer extension analysis of transcripts, a 32P-labeled probe (25,000–50,000 cpm) extending from positions +86 to +110 of the adenovirus E4 promoter was used. Purified reverse transcriptase products were analyzed on 8% polyacrylamide gels containing 7 M urea, and quantitated with a PhosphorImager (Molecular Dynamics, Sunnyvale, CA).

### Analysis of mutations in MAML1

MAML1 mutation data were downloaded from the Catalogue of Somatic Mutations in Cancer (COSMIC) database [26].

### Ubiquitination Experiments

HeLa cells were transfected with the different myc-tagged human mastermind constructs (MAML1-3) and HA-Ub using lipofectamine 2000 in 6-well dishes following the standard protocol. After 24 hours, the transfected cells were lysed in 1ml RIPA buffer (50 mM Tris-Cl, pH 7.9, 150 mM NaCl, 1% NP-40, 0.5% DOC, 0.1% SDS, 1X HALT protease cocktail, and 1% PMSF). Lysates were briefly sonicated and centrifuged to remove cell debris. The supernatants were transferred to new tubes and pre-cleared with Protein G PLUS-Agarose beads (Santa Cruz Biotechnology, Santa Cruz, CA, USA) for one hour at 4°C. Lysates were then centrifuged, supernatants transferred to a new tube, and 5 μg of anti-myc (9E10) antibody was added and incubated over night at 4°C on a nutator. The next day, protein G beads were added to each tube for one hour for immunoprecipitation. Beads were centrifuged and washed three times with RIPA buffer. After removal of the last wash, the beads were prepared for SDS-PAGE by adding 2X Laemmli buffer and boiling for 5 minutes prior to running on 4–12% Bis-Tris Gels (Invitrogen) or 10% SDS gels[27].

### Pulse Chase experiments to determine MAML1-3 Half-Lives

Transfections were performed for different Myc-tagged MAM constructs with lipofectamine 2000 in 6-well plates following the standard protocols described above. After 24 hours cells were treated with 150μg/ml of cycloheximide and extracts were collected every hour for 5 hours. The zero hour time point cells were untreated and collected with the 1 hour time point. Samples were processed as described above, except no IP was performed. Equal amounts of protein extracts were combined with 2X Laemmli buffer and boiled and loaded onto 4–12% Bis-Tris gels or 10% SDS Gels.

### SDS-PAGE and Western analysis

Samples were ran on various gels as described and transferred to PVDF membranes. Membranes were blocked with 5% non-fat dried milk in 1X Tris-buffered saline (20mM Trish pH 7.4, 500mM NaCl) with 0.1% Tween20 (TBST) for 1 h at room temperature and incubated overnight with primary antibody at 4°C. The following day, membranes were washed with TBST and probed with secondary antibody and developed using ECL (GE Healthcare, Piscataway, NJ, US) and images acquired on a LAS4010 imaging system (GE Healthcare, Piscataway, NJ, US).

### Quantitative Image Analysis to determine half-life of MAML1

Western blots for half-life studies, were quantified using ImageJ software (NIH, Bethesda, MD, USA) following recommend protocols. MAML1-3 proteins were normalized to either over-expressed CBF1 or native GAPDH.

### Statistical analyses

For comparison between two groups (Control vs. Treated), the data were analyzed using the two-sided student’s t-test with 95% confidence intervals reported. A *p*-value of *p* < 0.05 was regarded as statistically significant.

## Results

### MAML1 is ubiquitinated

**Fig 2:**
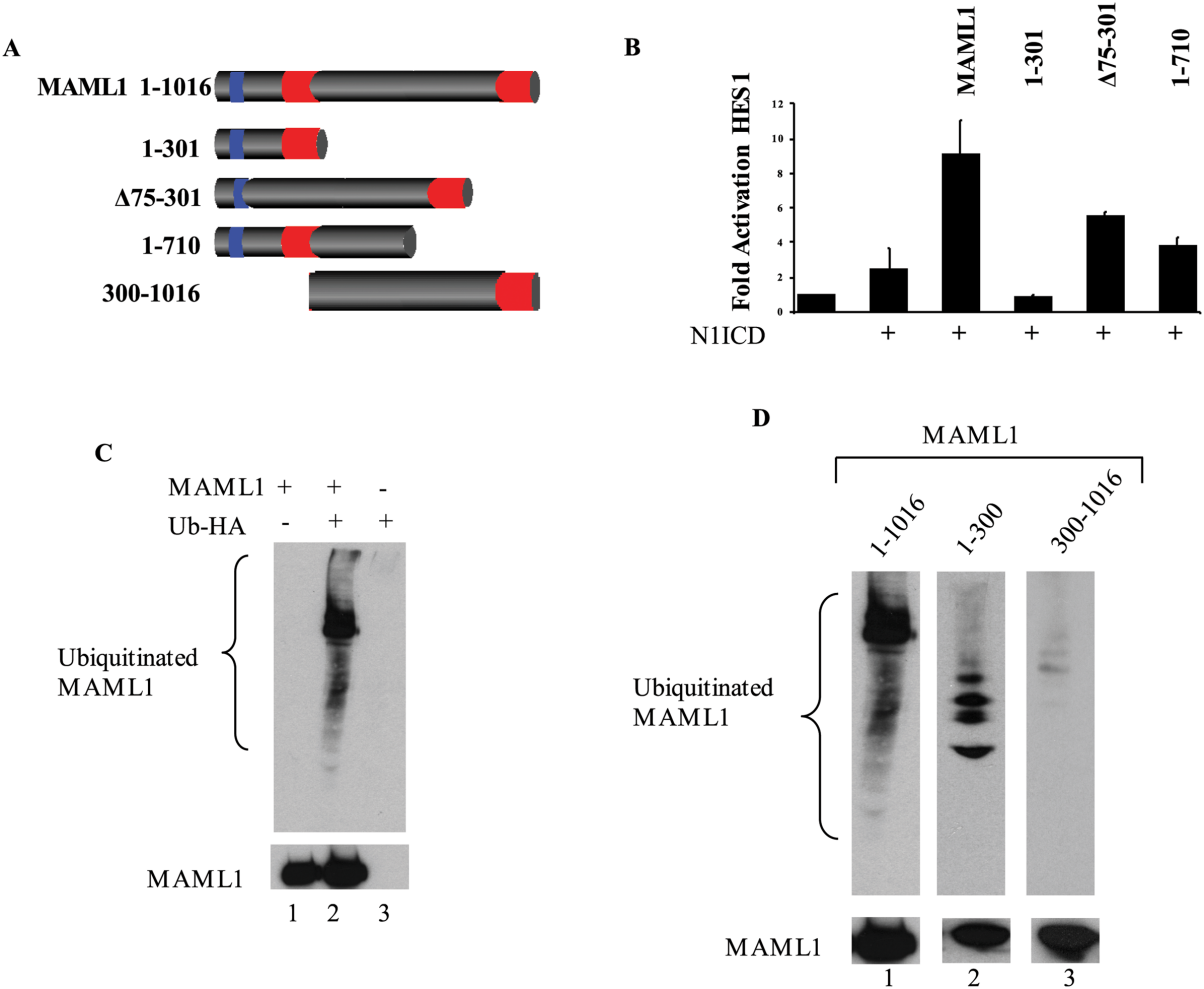
MAML1 is Ubiquitinated. (A) Cartoon depiction of MAML1 and the deletion constructs used in this study. MAML1 has been shown to have three domains, two acidic domains as indicated by red and one basic domain as indicated by blue. The acidic domain at the N-terminus (1–75 aa) has been shown to interact with the N1ICD. The central basic domain (75–300 aa) has been shown to interact with p300. The c-terminus domain has not been shown to interact with any proteins. (B) HES1 is activated by a combination of N1ICD with MAML1. Deletion of various domains of MAML1 show decreased reporter activity. Results are normalized to renilla luciferase and fold activation is compared to HES1 alone (n = 3). (C) MAML1 is ubiquitinated. MAML1 was overexpressed with HA-Ub. MAML1 was immunoprecipitated and western blots were performed to HA (Top blot) or MAML1 (lower blot) to show expression levels. (D) Ubiquitination of MAML1 occurs in the first 300 amino acids. MAML1 full-length, MAML1-1-300 or MAML1-300-1016 were expressed with HA-Ub and immunoprecipitated. Western blots were performed to ubiquitin (HA, top blot) or MAML1 constructs (Myc, bottom blot).

MAML1 has been previously shown to be a general co-activator of numerous transcription factors including MEF2C, p53, and Notch. Overexpression of MAML1 with N1ICD increased HES1 promoter activation compared to HES1 or HES1+N1ICD alone which is in agreement with previous reports (Fig 2B). MAML1 1–710, MAML1Δ75–301 and MAML1 1–301 (Fig 2A) had reduced activation of the HES1 promoter construct compared to MAML1. Further, MAML1 1–301 has also been shown to act as a dominant negative *in vivo* [7]. Our results suggested to us that either MAML1 could recruit other unknown factors that interact with the C-terminus or that the C-terminus could be modified. Previous reports have shown MAML1 to be phosphorylated [23] and acetylated [25]. Since phosphorylation has been shown to be required for ubiquitination of other transcription factors, we determined if MAML1 could be ubiquitinated. In Fig 2C, we show that the full-length MAML1 protein can be ubiquitinated. The ubiquitination was specific to MAML1 because overexpression of either Myc-MAML1 or HA-ubiquitin alone followed by IP to myc and western blot to HA did not show any detectable bands (Fig 2C, lanes 1 and 3).

### Mapping of MAML1 domains important for ubiquitination

We next tested deletion constructs of MAML1 to determine the region of ubiquitination. Western blot analysis of over-expressed myc tagged -MAML1, -MAML1-1-300, or -MAML1-300-1016 with HA-tagged ubiquitin in HeLa or HEK293 cells showed that MAML1 and MAML1-1-300 could be ubiquitinated. The MAML1-300-1016 was very weakly ubiquitinated (<0.5% of total MAML1 protein) (Fig 2C and 2D) suggesting that an unnatural deletion construction does not necessarily undergo ubiquitination in the cell.

**Fig 3:**
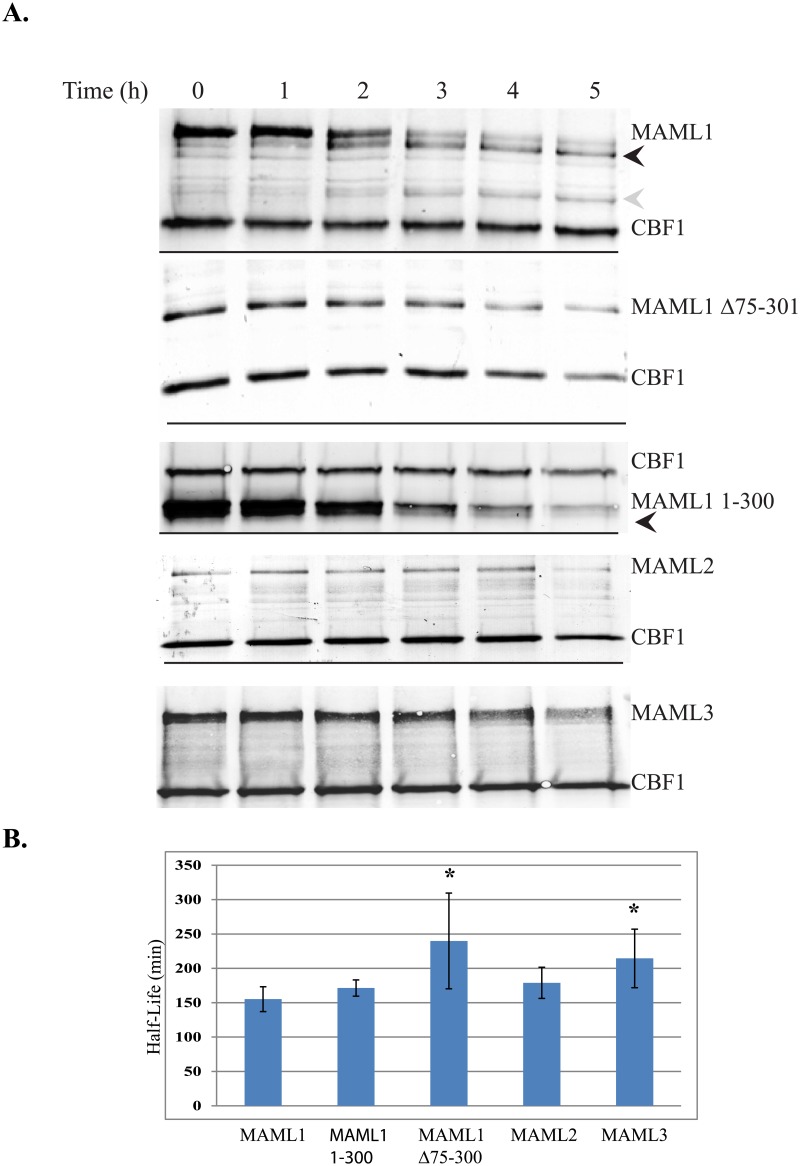
Half-life studies to MAML1-3 family members. (3A) MAML1, MAML1Δ75–301, MAML1-1-300, MAML2 or MAML3 were expressed with myc-tagged CBF1 in HeLa cells. Cells were treated with 150 μg/ml cycloheximide and cell extracts collected every hour for 5 hours. Western blots were performed to the myc-tag for the various proteins or to endogenously expressed GAPDH. Results shown are from representative blots form three different experiments. Black and gray arrowheads indicate degradation products that could be detected with time with the MAML1 and MAML1-300 constructs. (3B) ImageJ software was used to quantitate expression levels normalized to either CBF1 or GAPDH. The data is presented as the mean ± SD in bar graphs. A * indicates significantly different compared to MAML1 (p<0.05).

We have also conducted pulse chase experiments to determine the half-life of MAML1, MAML1 1–300, and MAML1Δ75–301. Myc-tagged vectors for the different constructs were overexpressed with myc-tagged CBF1 in HeLa cells. Representative blots are shown in Fig 3A of the results of the pulse-chase studies conducted for the different MAML1 proteins. Over time, an increase in a degradation product was detected for MAML1. The black arrowhead shows a degradation product that is immediately detected at the first time point and increases over time with maximum amounts seen between 2 and 3 hours. The gray arrowhead shows a degradation product that is seen early, but only begins to increase in amount after the three hour time point. This same trend could also be seen for the MAML1-1-300. The MAML1-1-300 is detected as a doublet as indicated by the black arrowhead. This doublet could be an early degradation product or a difference in the translation start site of the mRNA. However, both bands decreased over time. The MAML1Δ75–301 protein also decreased over time but at a slower rate than MAML1 or MAML1-1-300. We also could not detect any degradation products with the MAML1Δ75–301 protein. ImageJ software was used to quantitate and normalize the different MAML1 protein levels. We normalized to both the over-expressed CBF1 and to native GAPDH because both proteins were stable over time. Fig 3B shows a graph summarizing the calculated half-lives of the different MAML proteins with standard deviations. The half-life of MAML1 and MAML1-1-301 were 155 and 170 minutes respectively. Interestingly, this is the very similar to the half-life previously reported for the NICD when MAML1 is overexpressed with it (7). The half-life of the MAML1Δ75–301 was determined to be 240 minutes and was statistically different than MAML1 using a student’s t-test (p = 0.0161) regardless of normalization to CBF1 or GAPDH. Taken together this suggested to us that the degron region of MAML1 was found in the 75–300 region of the protein.

### Ubiquitination of MAML2 and MAML3 could not be detected

MAML1 is part of a family of MAML proteins found in mammals. There are three family members that have been identified (MAML1-3). MAML2 and MAML3 ubiquitination could not be detected consistently. We could detect MAML2 ubiquitination although it was very weak compared to the total amount of MAML2 protein present. We could not detect MAML3 ubiquitination in our system. We have tested for ubiquitination in both HeLa and HEK293 cell lines for MAML2 and MAML3 (data not shown) and have gotten similar results.

The level of ubiquitination was very low using a chemiluminescent detection agent and was hard to pick up above background levels at times. Because the ubiquitination was inconsistent, we used pulse-chase experiments to determine the half-lives of MAML2 and MAML3 and compare them to MAML1. We have also tested MAML2 and MAML3 for ubiquitination and have determined their half-lives to be 3 and 3.5 hours, respectively as shown in Fig 3B. However, using a student t-test, MAML2’s half-life was shown to not be statistically different than MAML1, whereas MAML3 was statistically different from MAML1 (p = 0.0112).

### Mapping lysine residues that are important in ubiquitination of MAML1

**Fig 4:**
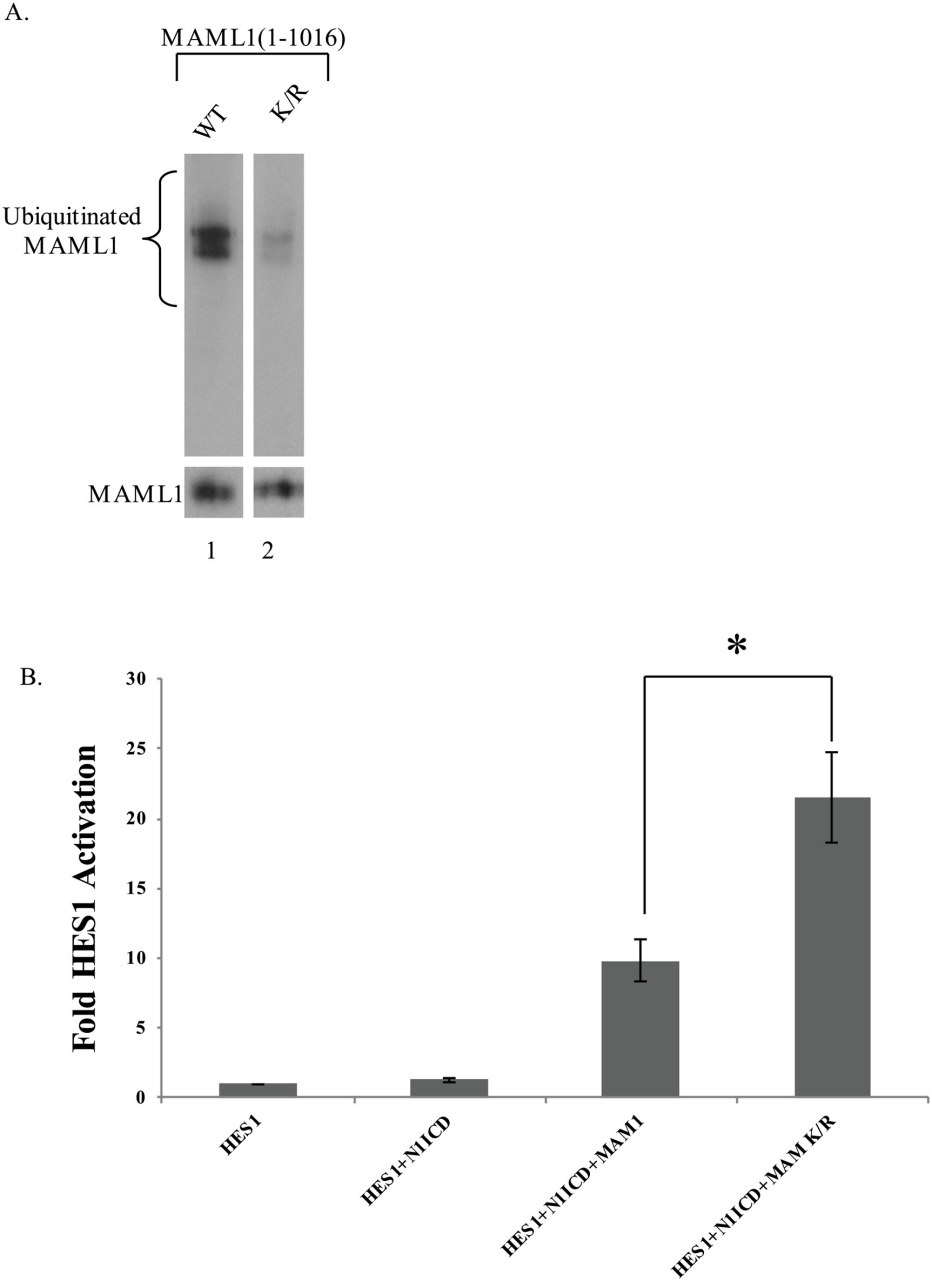
Identification of conserved lysine residues responsible for ubiquitination of MAML1. (A) MAML1 was mutated at various lysine residues as described in text. Myc-tagged MAML1 Wild type (WT) or Mutant (K/R) was expressed with HA-tagged ubiquitin and immunoprecipitated to the myc tag. Western blots were performed to HA (upper blot) or myc (lower blot) for loading control. Ubiquitination was decreased by greater than 95% in the mutant compared to wild type MAML1. (B) MAML1 K/R mutant activates a HES1 reporter construct stronger than MAML1 WT. HeLa cells were co-transfected with HES1-luciferase reporter construct, renilla luciferase, N1ICD, and either MAML1 WT or MAML1K/R. HES1-Luciferase activity assays were performed and results shown are the mean ± SD (n = 4). *, p<0.05.

MAML1 contains three well characterized domains (Fig 2A): the N-terminal acidic domain which contains the first 75 amino acids and binds to NICD, the basic domain (75–301aa) which binds to p300 and the c-terminal acidic domain which has not been shown to interact with a protein. Our results suggested that ubiquitination might be occurring around the 75–300 region because deletion of this region resulted in an increase in half-life and ubiquitination could not be detected. Therefore, we began mutating a series of lysine residues in MAML1 and testing for a decrease in ubiquitination. As shown in Fig 4A, in order to remove >95% of the ubiquitination from the over-expressed protein, we needed to mutate 8 lysine residues to arginine (K112, 178, 188, 189, 405, 407, 639, 822R) and have designated this construct as MAML1K/R for simplicity. Both the MAML1 and MAML1K/R proteins were over-expressed at the same level indicating that the decrease in ubiquitination was due to mutation of K residues to R residues and not overall change in recombinant protein levels.

**Fig 5:**
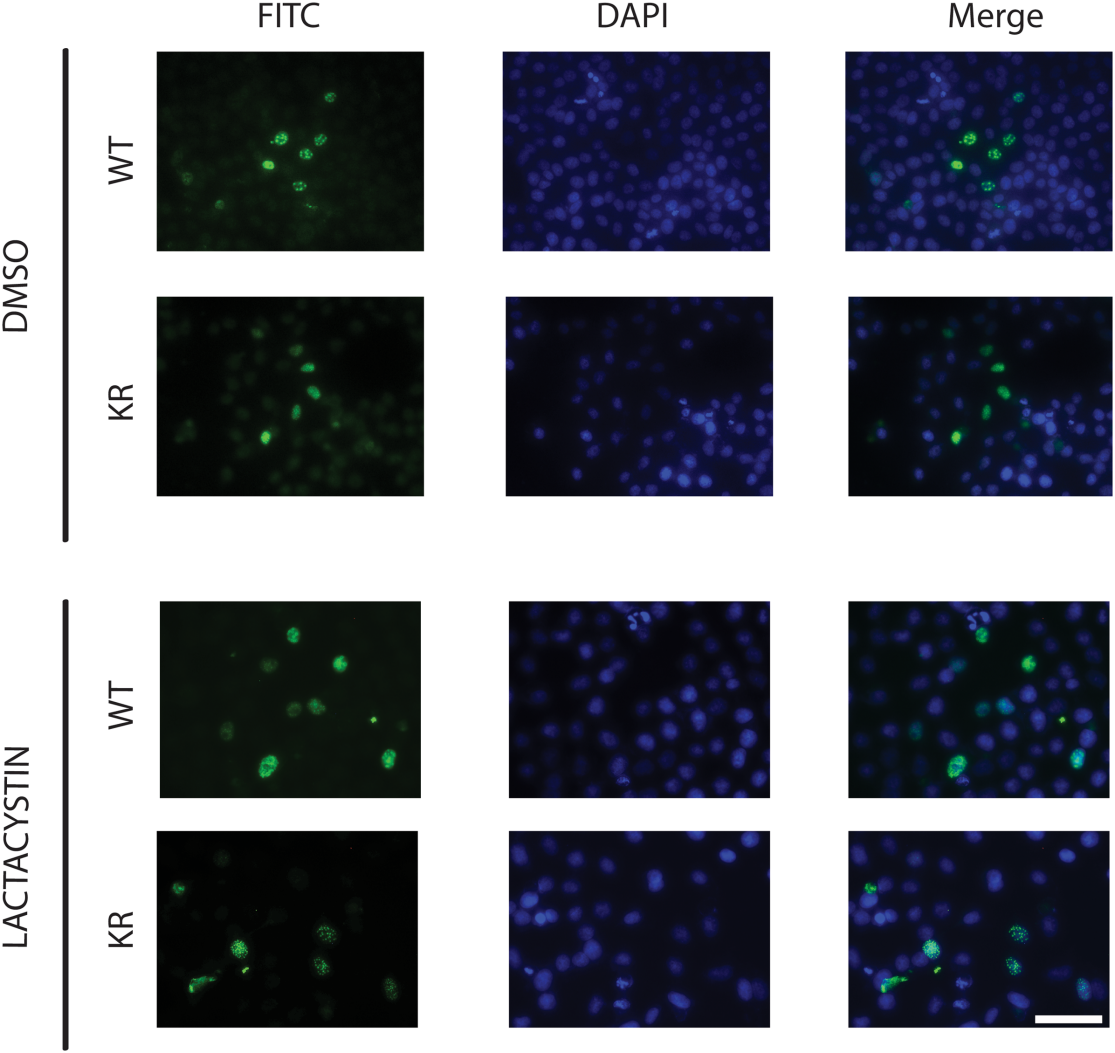
Localization of MAML1 in HeLa cells. MAML1 was mutated at various lysine residues as described in text. Myc-tagged MAML1 Wild type (WT) or Mutant (KR) was expressed in HeLa cells. Twenty-four hours post-transfection, cells were either treated with DMSO or lactacystin (10 μM) and left for an additional 24 hours. Cells were then fixed in 4% paraformaldehyde and processed for immunocytochemistry. Both MAML1 and MAML1 KR mutant localize to nuclei in discrete foci. Images show MAML1 WT and KR with FITC stain alone, DAPI stain to detect nucleus along, or merged to show overlap. Scale Bar is 50 μm.

We tested the MAML1K/R protein in our HES1 reporter gene assay to determine if mutation of the lysine residues had any effect on transcriptional activation. As shown in Fig 4B, overexpression of MAML1 with N1ICD resulted in an increase in promoter activation compared to HES1 or HES1+N1ICD alone. However, overexpression of MAML1K/R with N1ICD had a significantly higher response (p<0.05) compared to the wild type MAML1. We also looked at the localization of MAML1K/R compared to MAML1. MAML1 has previously been shown to localize to the nucleus in discrete foci [28]. Both proteins localized to the nucleus in foci. The foci were unaffected by treatment with lactacystin (compared to DMSO treatment). This data suggests that the mutations in the lysine residues appear to only be important in ubiquitination and do not alter any other functionality (Fig 5).

Since we saw different half-lives for MAML2 and MAML3 compared to MAML1, it prompted us to determine if the lysine residues that we identified in MAML1 were also present in MAML2 and MAML3. The difference in ubiquitination levels could be explained by different number of lysine residues being ubiquitinated. An alignment of MAML1-3 proteins using CLUSTALW indicated that the K residues found in MAML1 were not conserved in MAML2 or MAML3 with the exception of K162 in MAML2 (K112 in MAML1) and K190 in MAML3 (K178 in MAML1) (data not shown).

### MAML1 ubiquitination is stimulated by p300 and inhibited by N1ICD

**Fig 6:**
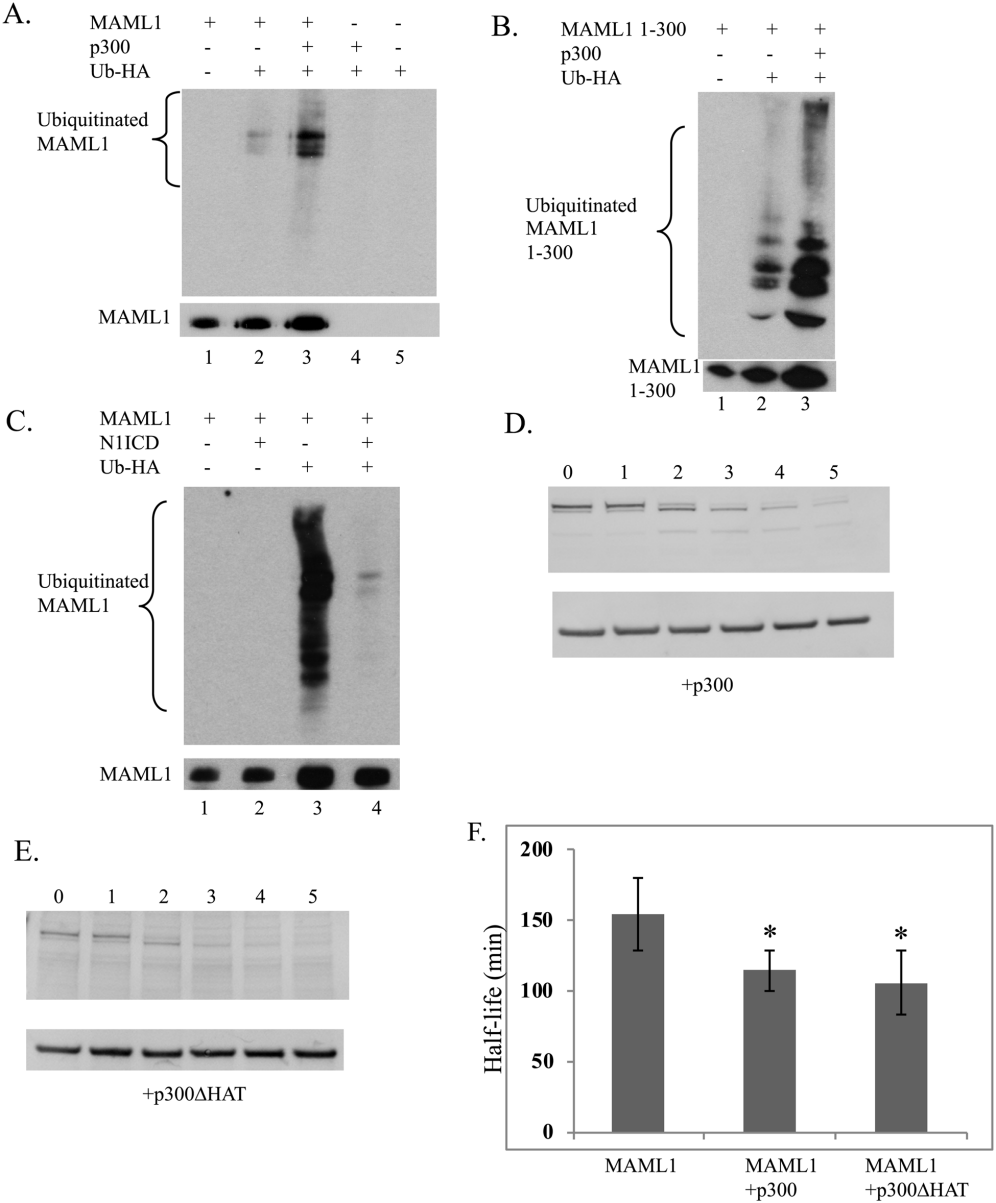
MAML1 ubiquitination is stimulated by p300 and inhibited by NICD. (A, B, C) Western blot analysis for ubiquitination of myc-tagged MAML1 or MAML1-300 was performed in the presence or absence of p300 (6A and 6B) or N1ICD (6C). MAML1 proteins and HA-Ub were expressed and immunoprecipitated as described in the text in the presence or absence of p300 or NICD. Prior to IP, a sample was taken to monitor total protein levels for MAML1. Western blots were performed to either the IP for ubiquitination or total extract was blotted as a loading control for MAML1 expression. p300 stimulated the ubiquitination of both MAML1 and MAML1 1–300 (compare lanes 2 and 3 in 6A and 6B) whereas NICD decreased the amount of ubiquitination of MAML1 (compare lanes 3 and 4 in 6C). p300 and p300ΔHAT stimulated degradation of MAML1 (6D and 6E). MAML1 was overexpressed with p300 or p300ΔHAT and pulse-chase experiments performed as previously described (See Fig 2 text). Western blots were performed and results were normalized to GAPDH levels and results are shown ± SD (n = 4). (6F) The half-life of MAML1 is significantly decreased in the presence of p300 (p = 0.025) or p300ΔHAT (p = 0.0146).

We next conducted biochemical experiments to look at how expression of other protein binding partners would affect the ubiquitination of MAML1. Overexpression of Myc-tagged MAML1 with Flag-tagged p300 and HA-Ub resulted in an increased ubiquitination of MAML1 compared to MAML1 and HA-Ub alone (Fig 6A compare Lanes 2 and 3). The p300 stimulated ubiquitination also occurred with the MAML1 1–300 construct (Fig 6B compare lanes 2 and 3). Interestingly, overexpression of MAML1 with NICD resulted in a decrease in the ubiquitination of MAML1 (Fig 6C, compare lanes) suggesting that the interaction with the NICD blocks the ability of MAML1 to be ubiquitinated.

Because p300 stimulated ubiquitination of MAML1, we performed pulse-chase experiments with overexpression of MAML1 and p300 compared to MAML1 alone as previously described in methods to determine if there was a difference in the half-life (Fig 6D). Overexpression of p300 decreased the half-life of MAML1 to 2 hours compared to 2.5 hours for MAML1 alone (Fig 6F) and this change was significant (p = 0.025) as determined by a student’s t-test.

Interestingly, the p300 mediated increase in ubiquitination does not require the histone acetylase (HAT) activity of p300. Using a p300ΔHAT construct, MAML1 degradation was also significantly faster (p = 0.0146) compared to MAML1 alone (Fig 6E and 6F).

**Fig 7:**
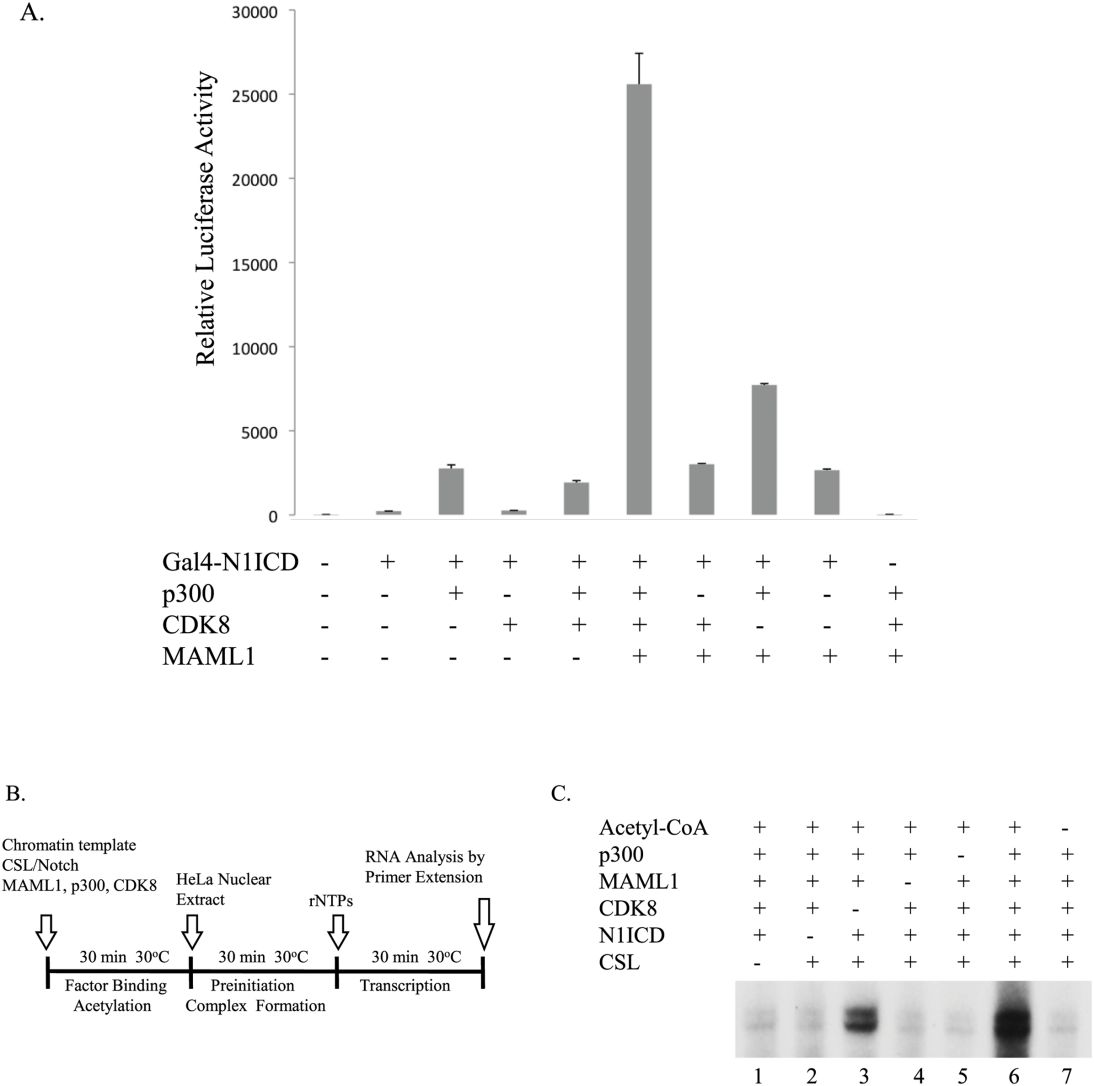
MAML1, CDK8 and p300 cooperatively stimulates Notch activity. **(A)** HEK293 cells were co-transfected with a GAL4 luciferase gene and vectors expressing GAL4-Notch, MAML1, p300 and CDK8, as indicated in the figure. The data is presented as mean ± SD (n = 3). MAML1, p300, and CDK8 stimulate transcription in the presence of GAL4-Notch (*, p<0.0001). **(B)** Schematic of the *in vitro* transcription assay using chromatin templates. **(C)** Chromatin templates containing CSL binding sites were incubated with N1ICD, MAML1, CDK8, p300 and acetyl-CoA, as indicated.

We further investigated the functional interplay between Notch and the Notch coactivators MAML1, p300 and CDK8 by cotransfecting HEK-293 cells with a plasmid containing five GAL4 sites upstream of a luciferase gene and plasmids expressing GAL4-N1ICD, p300-HA, CDK8-FLAG and MAML1. We found that although MAML1, p300 and CDK8 enhanced Notch activity when co-expressed alone with Notch, they more potently enhanced Notch transcription in each other’s presence compared to Notch alone (Fig 7A) suggesting a synergy (p<0.0001). In addition, we utilized a cell-free transcription system with chromatin templates and the recombinant proteins Notch, CSL, p300, CDK8 and MAML1 purified from insect cells, as previously described [29]. The CSL template, containing the E4 promoter and binding sites for CSL, was reconstituted with HeLa core histones by using purified recombinant Drosophila Acf-1, ISWI, and NAP1 proteins as previously described [23, 30]. Assays were performed according to the protocol indicated in Fig 6B with HeLa nuclear extract as a source of general transcription factors. As indicated in Fig 6C, a significant level of transcription was observed when the chromatin template was incubated with CSL, Notch, MAML1, p300 and acetyl-CoA (lane 3), and this activity was completely dependent upon the presence of p300 (lane 5), acetyl-CoA (lane 7) and Notch1 IC (lane 2). Addition of CDK8 increased the activity observed with CSL, Notch, MAML1, p300 and acetyl-CoA approximately 4-fold (lane 6 versus lane 3), and this activity was likewise completely dependent upon CSL and Notch (lanes 1 and 2), MAML1 (lane 4) and p300 (lane 5). These results clearly indicate a functional cooperativity between the Notch coactivators MAML1, p300 and CDK8.

### MAML1 is mutated in various cancer cell lines

We screened the Catalogue of Somatic Mutations in Cancer (COSMIC) database for mutations in MAML1 [26]. Nonsense and frameshift mutations are summarized in Table 1 from the various cancer cell lines. The table lists the primary tissue, cell line, amino acid location and corresponding nucleotide. Although we did not identify any lysine residues mutated in any of the cell lines, we cannot rule out if any of the mutations identified could possibly affect ubiquitination such as through binding of the ubiquitin ligase or some other function.

**Table 1:** Summary of mutations identified in various cancer cell lines. NS corresponds to non-specific cell lines that have not been assigned to a primary tissue.

Primary tissue	Cell line	AA Mutation	CDS Mutation
Large intestine	CW-2	p.P263S	c.787C>T
	CW-2	p.P435L	c.1304C>T
	LoVo	p.P475H	c.1424C>A
	KM12	p.G531W	c.1591G>T
	GP5d	p.N786D	c.2356A>G
Skin	SK-MEL-2	p.S838F	c.2513C>T
	CP67-MEL	p.S882F	c.2645C>T
Haematopoietic and lymphoid	Jurkat	p.A378V	c.1133C>T
	ALL-PO	p.I402M	c.1206C>G
	ST486	p.R668C	c.2002C>T
	GDM-1	p.G513fs	c.1537delG
	HAL-01	p.P771Q	c.2312C>A
	KARPAS-45	p.A908V	c.2723C>T
	KMOE-2	p.L1009M	c.3025T>A
Lung	A427	p.N153H	c.457A>C
	NCI-H2085	p.N786S	c.2357A>G
Central nervous system	CAS-1	p.P354R	c.1061C>G
Ovary	KGN	p.N358K	c.1074T>A
	OVCAR-8	p.Q617L	c.1850A>T
Stomach	GCIY	p.N358K	c.1074T>A
Soft-tissue	SK-UT-1	p.L258P	c.773T>C
Salivary gland	HO-1-N-1	p.S151F	c.452C>T
Endometrium	COLO-684	p.A366T	c.1096G>A
NS	DND-41	p.H725R	c.2174A>G
	FU97	p.N358K	c.1074T>A
	MFE-319	p.A946V	c.2837C>T
	OV-90	p.R710Q	c.2129G>A
	SNU-1040	p.A403T	c.1207G>A
	SNU-1040	p.A655T	c.1963G>A
	SNU-1040	p.G697S	c.2089G>A
Vulva	CAL-39	p.G124S	c.370G>A

## Discussion

Regulation of gene transcription is important for the maintenance of normal cellular processes. Failure to correctly regulate gene expression can lead to disease. For example, stabilization of the NICD in the nucleus causes hyperactivation of various genes including c-myc and has been shown to be involved in leukemiogenesis [14]. Therefore, tight control of gene regulation must occur. One way a cell can do this is by making use of the proteolytic activities of the ubiquitin proteasome system. Many transcriptional activators have been identified to be ubiquitinated thereby shutting transcription off and resetting genes for future activation (reviewed in [21]). To date, very few transcriptional co-activators have been shown to be ubiquitinated. Most notably, PGC1-α, a coactivator of the cellular energy metabolism pathway, who interacts with PPAR-γ was shown to be ubiquitinated to control intracellular levels [31]. Further, SRC-3, a histone acetyl transferase implicated as an oncogene, has also been shown to be ubiquitinated to maintain low levels of the protein [32]. Here we report the co-activator, MAML1, to also be ubiquitinated.

MAML1 may be ubiquitinated and this ubiquitination leads to degradation. We have mapped residues important in ubiquitination to lysines that span the region from aa 100–800. Only when mutating a total of 8 lysine residues to arginine (K112, 178, 188, 189, 405, 407, 639, 822R) together do we decrease the levels of ubiquitination of MAML1. Mutation of individual lysines had no effect on protein stability (data not shown), perhaps due to large accumulation of the overexpressed proteins. Further, deletion of the MAML1 75–301aa domain stabilized the half-life of the protein compared to MAML1. This is also the same region that has been shown to bind to p300 [7]. We showed that over-expression of p300 with MAML1 increased the ubiquitination and decreased the half-life compared to MAML1 alone. Since deletion of the p300 binding domain leaves four lysine residues in the protein (405, 407, 649, and 822), we speculate that perhaps p300 may help recruit an unidentified ubiquitin ligase and with removal of this domain, the ubiquitin ligase is not able to be recruited to ubiquitinate MAML1. Interestingly, the MAML1 300–1016 protein was also weakly ubiquitinated (Fig 2D) and although we did not determine the half-life of this protein, we anticipate that it would also be stable because it would be unable to interact with p300. The N1ICD binding region has been mapped to the first 75 aa of MAML1 and since our data suggests that N1ICD binding promotes stabilization of MAML1, we speculate that this might be occurring through preventing the ubiquitin ligase from interacting with MAML1. However, further studies would be need to be conducted as well as identifying a ubiquitin ligase.

MAML1 has also previously been shown to be post-translationally modified by other mechanisms including phosphorylation [23] and acetylation [25]. It has been proposed the acetylation leads to increase in transcriptional output. However, given that p300 is also able to increase the ubiquitination of MAML1 *in vivo* and it does so in the absence of a HAT domain, it remains unclear the exact relationship between these two modifications. Other transcriptional activators, including NICD, require phosphorylation as a mechanism by which the ubiquitin ligase is recruited for ubiquitination to proceed [21].

Interestingly, we were only able to detect strong ubiquitination of MAML1. We detected weak ubiquitination of MAML2 and could not detect ubiquitination of MAML3 in our system. We also noticed that MAML2 and MAML3 have different half-lives than MAML1 although the half-lives were not shown to be statistically significant. An alignment of the MAML1-3 proteins revealed that the lysine residues found in MAML1 to be important for ubiquitination are not conserved in MAML2 and MAML3 with the exception of the residues mentioned in the results. Taken together these results suggest that MAML2 and MAML3 may be regulated differentially compared to MAML1.

The MAML1K/R mutant protein was unable to be ubiquitinated and we were also able to see an increase in transcriptional output from this protein in a HES1-Luciferase assay system. We attribute the increase in reporter gene activity to be due to an increased amount of MAML1K/R protein present in the cells or that the ternary complex remains more stable on the DNA. We did not determine if the MAM1K/R protein was able to stimulate degradation of the N1ICD as previously reported (8). However, further investigation is needed to determine the relationship between the MAM1K/R protein and transcription.

We screened the Catalogue of Somatic Mutations in Cancer (COSMIC) database for mutations in MAML1 [26]. Although we did not find any mutated lysine residues, numerous mutations were found. Many of these mutations were found in the C-terminal region of MAML1. The C-terminal domain of MAML1 is required for strong transcriptional activation although the mechanism of action has not been identified. Many of the mutations identified also occurred in proline residues. Prolines are known to be important for protein folding. We speculate that these mutations could affect protein stability [33]. None of the mutations that have been identified suggested to us that they were activating (i.e. increase protein activity). Nonetheless, some of these mutations could prevent an ubiquitin ligase from binding to MAML1 to ubiquitinate the protein. This would then increase protein stability and we would suspect increase transcriptional output from target genes. Further investigation is needed to determine the effects these mutations may have on MAML1 activity and what role they may play in neoplastic transformation.

To date the only mutations in MAM family members that have been shown to potentially play a role in oncogenesis involve the fusion of the C-terminal domain of MAML2 with N-terminal domain of the CREB regulator MECT1. This fusion protein is able to interact with CREB and constitutively activate CREB-dependent genes in salivary gland tumors [34].

Ubiquitination of transcription factors has been shown to occur through a phosphorylation mechanism, whereby the transcription factor is first phosphorylated to recruit a ubiquitin ligase. This has been shown to be true for Notch, GAL4, and cMyc amongst other transcription factors [21]. We tested CDK8 and GSK3Beta for their ability to stimulate degradation of MAML1, but we did not see any changes in the ubiquitination level (data not shown). Interestingly CDK8, together with p300 and MAML1, synergizes to stimulate transcriptional activation. However, since acetylation of MAML1 does not appear to have an effect on ubiquitination, understanding the relationship between acetylation and phosphorylation still remains unresolved.

**Fig 8:**
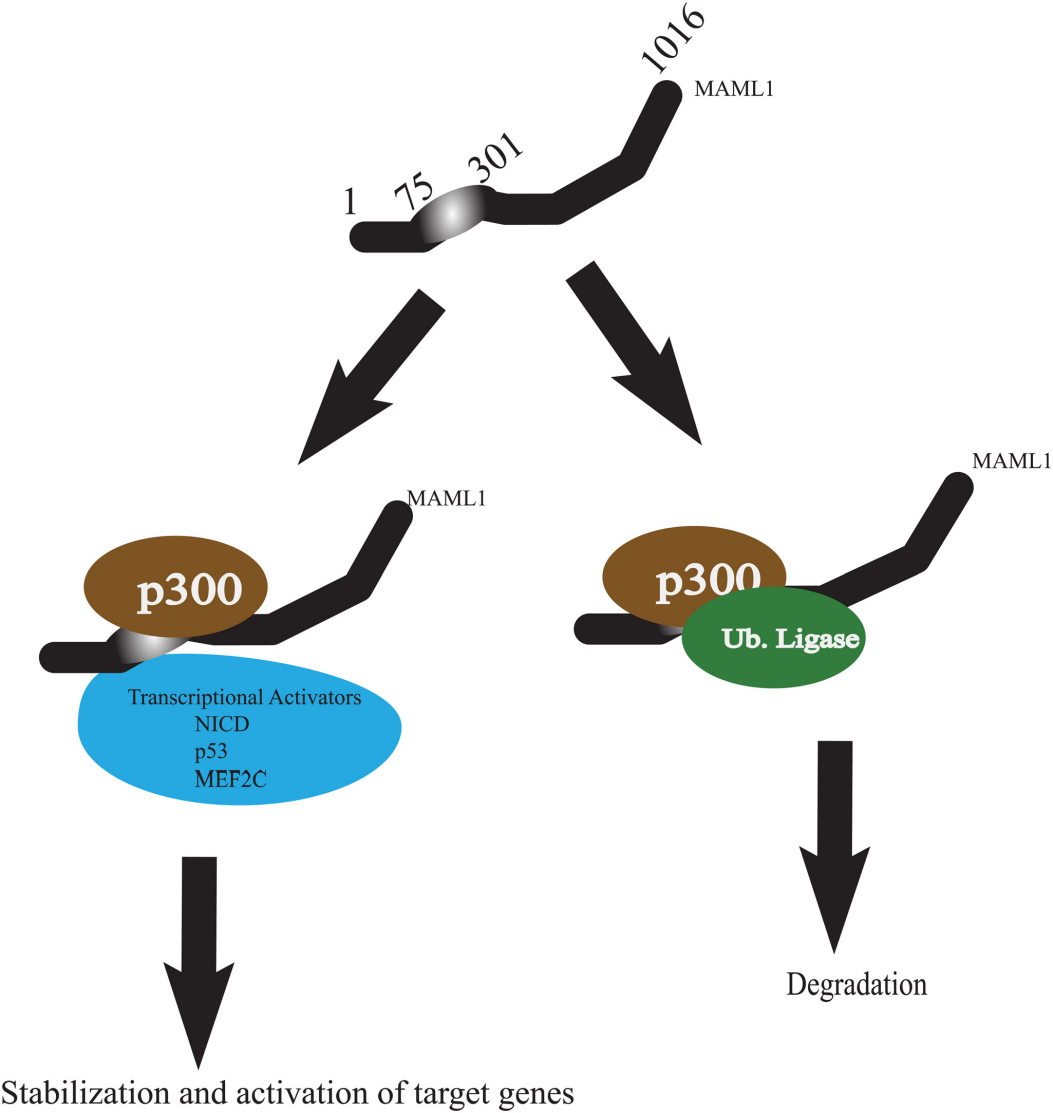
Proposed Model for Importance of MAML1 Ubiquitination. In the absence of activators (MEF2C, p53, NICD), MAML1 levels must remain low in the cell in order to prevent nonspecific transcriptional activation. MAML1 interacts with p300 to recruit an ubiquitin ligase to ubiquitinate MAML1 resulting in degradation. When activators are present, MAML1 is stabilized allowing co-transcriptional activation of target genes to occur.

We propose a mechanism to account for the ubiquitination and degradation of MAML (Fig 8). In the absence of a transcriptional activator (NICD, p53, MEF2C), MAML1 levels need to remain low in the cell in order to prevent non-specific activation of genes. Increased levels of MAML1 in the nucleus in the absence of one its activators could lead to increased non-specific interaction with other transcription factors and subsequent recruitment of p300 to acetylate histones at target genes. Only when a transcriptional activator is present would MAML1 levels become stabilized allowing proper transcriptional complex formations in the nucleus.
